# Incidence and predictors of neuropsychiatric manifestations following a traumatic brain injury at referral hospitals in Dodoma, Tanzania: A protocol of a prospective longitudinal observational study

**DOI:** 10.1371/journal.pone.0311091

**Published:** 2024-10-28

**Authors:** Suluma Aslan, Azan Nyundo

**Affiliations:** 1 Department of Psychiatry and Mental Health, School of Medicine, The University of Dodoma, Dodoma, Tanzania; 2 Mirembe National Mental Health Hospital, Dodoma, Tanzania; 3 Department of Internal Medicine, The Benjamin Mkapa Hospital, Dodoma, Tanzania; PLoS ONE, UNITED STATES OF AMERICA

## Abstract

**Introduction:**

Traumatic Brain Injury (TBI) is any injury to the brain resulting from an external force leading to complications. TBI affects 27–69 million people yearly, with high incidence in low- and middle-income countries (LMICs), mainly attributed to motor traffic accidents. Within three to six months following moderate-to-severe TBI, the relative risk of any psychiatric disorder is significantly higher than in the general population. Post-TBI neuropsychiatric disorders include depression with a prevalence of up to 53%, apathy up to 72%, Posttraumatic stress disorder (26%), anxiety (9%), manic symptoms (5–9%) and psychosis (3 to 8%). This study aims to determine the incidence and predictors of post-TBI neuropsychiatric manifestations among patients admitted at Referral hospitals in Dodoma.

**Methods and analysis:**

This is a prospective longitudinal observational study in which patients admitted with moderate to severe TBI will be recruited. Patients will be followed for six months, the diagnostic MINI International Neuropsychiatric Interview (MINI) tool will be used to assess TBI, and the severity and progression of symptoms will be evaluated using PHQ-9 for depressive symptoms, GAD-7 for anxiety symptoms, PCL-5 for Posttraumatic Stress Disorders (PTSD), MoCA for cognitive assessment, AES for apathy and YMRS for manic symptoms at one, three and six months. Logistic regression will be analysed to determine the association between predictors and neuropsychiatric outcomes.

**Conclusion:**

Given the dearth of understanding of the burden of neuropsychiatric complications and associated outcomes in sub-Saharan Africa, the study will shed light on the incidence and factors contributing to post-TBI neuropsychiatric complications and, thus, provide a platform for further research and design of necessary interventional programs for the population at risk.

## Introduction

Of 7.3 billion people, 27 to 69 million are expected to suffer from TBI annually [1, 2], the majority of whom reside in LMICs, comprising 85% of the world’s population and roughly 90% of all injury-related fatalities [3]. TBI is predicted to significantly burden Africa, with around 6 and 14 million additional cases by 2050 [4] primarily attributed to motor traffic accidents, also observed at Muhimbili Orthopedic Institute (MOI) in Dar es Salaam, Tanzania, where traffic accidents are the most common cause of TBI [5].

The severity of TBI is determined by the Glasgow Coma Scale (GCS), with a maximum score of 15 scores range categorized as mild (13–15), (9–12) as moderate and (≤ 8) as severe, the scale combines eye, motor, and visual responses to rate levels of consciousness [6].

TBI can result in several neuropsychiatric symptoms (NPS), with an estimated annual prevalence of up to 53% [7]; depression is the primary cause of disability post-TBI [8], and a prior history of depression and alcohol dependence significantly increases the risk for post-TBI depression [7].

A 20 to 72 per cent prevalence makes apathy among the most common neuropsychiatric outcomes following traumatic brain injury [9]. Based on the Glasgow Outcome Scale, severe disabilities after TBI are likely to exhibit apathetic behaviours [10]. Closely mimicking apathy are the cognitive deficits caused by primary brain injury or secondary impairments, including sleep disturbances [11]. Potential causes of cognitive impairment post-TBI include pre-TBI cognitive impairment, TBI severity, involvement of specific brain regions, the damage mechanism, genetics, and advanced age [12].

Other neuropsychiatric manifestations post-TBI include anxiety disorders, psychosis, manic symptoms and neurocognitive disorders [11]. The rate of anxiety disorder ranges between 19 and 50 per cent reported at distinct post-injury durations [13, 14]. During the six months following TBI, 26.8% present with significant PTSD symptoms, which are associated with functional impairment, post-concussive symptoms, cognitive decline related to poor performance in visual processing and mental flexibility and overall decline in life satisfaction [15]. While PTSD is more likely to result from psychologically upsetting events, such as memory of the traumatic events and early posttraumatic stress symptoms [16], neuroanatomical factors, including Hippocampus volume loss, amygdala hyperactivity, and hypoactivity in the ventromedial prefrontal cortex(vmPFC) are all associated with PTSD after TBI [17].

A prevalence of 5–9% is reported for Manic symptoms [18], with focal brain injuries in the frontal lobe being implicated in the genesis of post-TBI manic symptoms [19]. Compared to the general population, newly diagnosed Bipolar I Disorder are 1.5 times more likely to have experienced a head injury in the past five years [20].

As for psychotic symptoms, 3–8% of TBI patients get psychotic illnesses after their injury [21]. Risk factors for posttraumatic psychosis include male gender, pre-injury neuropsychiatric problems, family history of schizophrenia, the severity of the brain damage, involvement of the temporal and frontal lobes, left hemisphere laterality, EEG abnormalities, posttraumatic epilepsy, and pre-TBI cognitive impairment [22].

Although the incidence and predictors of neuropsychiatric manifestations of TBI are widely reported elsewhere, research is scarce in sub-Saharan settings. To the best of our knowledge, there are only a few published studies in Africa regarding post-TBI neuropsychiatric manifestations [23, 24], and none has been published in Tanzania. Given the severity of neuropsychiatric disorders and related short and long-term complications, early diagnosis and prompt intervention can prevent long-term disability from TBI [25, 26] through specific tailor-made recovery and rehabilitation programs suited to the patient’s needs [23].

The study aims to determine cumulative incidence and factors associated with neuropsychiatric manifestations after TBI among patients admitted at referral hospitals in the Dodoma region of Tanzania.

### Specific aims

1. To describe baseline neuropsychiatric disorders at one month following TBI in referral hospitals in Dodoma, Tanzania.
2. To determine factors associated with baseline neuropsychiatric manifestation in TBI patients in referral hospitals in Dodoma.
3. To determine the cumulative incidence of neuropsychiatric disorders at three and six months following TBI in referral hospitals in Dodoma, Tanzania.
4. To determine predictors of a neuropsychiatric disorder at three and six months following TBI in referral hospitals in Dodoma, Tanzania.

## Materials and methods

### Study design

This is a prospective longitudinal observational study.

### Study area

The study will be conducted at the referral hospitals in Dodoma: The Benjamin Mkapa Hospital (BMH) and Dodoma Regional Referral Hospital (DRRH). Dodoma is the capital city of Tanzania, located in the central part of the country, serving approximately three million people from Dodoma and nearby regions [27]. The bed capacities of the two hospitals are 400 and 480 for DRRH and BMH, respectively. Based on local data, these hospitals receive 7 to 10 patients with moderate to severe TBI per week. The hospitals have surgeons and neurosurgeons qualified for procedures needed after TBI. In the case of neuroimaging, a CT scan is performed in both hospitals and MRI is performed at BMH. Patients will be found in both hospitals’ surgical wards, neurosurgical wards and ICUs.

### Study population

This study involves all patients aged 18 years and above admitted at DRRH or BMH after moderate or severe TBI within seven days after TBI. Some patients are brought directly to these hospitals, and others are referred from district hospitals surrounding the Dodoma region.

### Inclusion criteria

1. Patients with age between 18 years and above who are admitted after their first traumatic brain injury
2. Patients who scored less than 13 in GCS
3. Patients with the capacity to provide informed consent or close family member or custodian for those who cannot.

### Exclusion criteria

1. Patients with a known CNS diagnosis, such as CNS tumours or any space-occupying brain lesion.
2. Patient with a recent history of severe stroke.
3. Patient with a history of psychotic disorders like Schizophrenia spectrum disorder before TBI.

### Sample size

We use Krejcie and Morgan formula to calculate the sample as follows;

$$\begin{array}{ll} \text{N} & =\frac{\text{N}.X^{2}.\text{p}.\left(1-\text{p}\right)}{X^{2}.\text{p}.\left(1-\text{p}\right)+e^{2}.\left(\text{N}-1\right)}\\ & =\frac{171.{\left(2.576\right)}^{2}\text{X}\;0.5\left(1-0.5\right)}{{\left(2.576\right)}^{2}X\;0.5\left(1-0.5\right)+{\left(0.01\right)}^{2}\text{X}\left(171-1\right)}\\ & =170\left(\text{with}\;20\text{\%}\;\text{attrition}\right)\\ & =204 \end{array}$$

Where n—Calculated sample; N–Population = 171 from [23] X—Normal standardized variable associated with the confidence level = 2.576; p-True probability of the event = 50%(because there was no prev. literature) e—sample error = 0.01.

### Sampling procedure

A consecutive sampling method will be used to recruit all the patients who meet inclusion criteria found in the surgical ward, neurosurgical ward, and ICU of DRRH and BMH. This will be done by taking all the available patients who fit the inclusion criteria until the desired sample is reached.

### Data collection

#### At baseline

Patient’s information will be entered into an Open Data Kit (ODK) using an Android mobile device, where social demographic characteristics, including age, sex, education level, and occupation, will be collected. A history of substance use and history of being intoxicated with alcohol prior to TBI will also be documented.

TBI clinical presentation will be documented, including the severity of the TBI measured by using the Glasgow Coma Scale (GCS), which includes moderate (9–13) and severe (8–3) immediately following an injury [6], duration of loss of consciousness(LOC) [12] and duration of post traumatic amnesia [12] Associated clinical presentations, including seizures, speech difficulties, confusion and laterality, will be recorded. The nature of the trauma will be classified as open or closed, penetrative or non-penetrative. The cause of injury will be recorded as Road Traffic Accident (RTA) (motorcycle, motor vehicle), falls, and being hit by objects or sports. Type of TBI lesions, including haematoma, contusion, intracerebral haemorrhage, subarachnoid haemorrhage, and diffuse axonal injury, will be documented after being confirmed by CT scan or MRI as part of clinical workup. These patients will be followed up and assessed for neuropsychiatric manifestations at one, three and six months.

#### At one month

Baseline assessment of the neuropsychiatric manifestations, including Depression, Generalized Anxiety, Bipolar, Apathy, Psychosis and Neurocognitive symptoms, will be assessed using standardized diagnostic and screening tools (See the data collection instrument).

#### At three and six months

The cumulative incidence of neuropsychiatric disorders will be assessed. The onset of a new neuropsychiatric disorder will be identified by using the screening tools used at baseline. The tools will identify significant change in the severity of neuropsychiatric symptoms, defined as either a significant reduction in symptoms that the criteria is no longer met or an increase in symptom severity that a new diagnosis is currently met.

### Outcome measurements

#### Definition and measurement of variables

*Aim 1 study variables*. These variables address baseline neuropsychiatric disorders at one month following TBI. The presence of neuropsychiatric manifestations will be measured using screening and diagnostic tools (Table 1).

**Table 1:** Description of baseline neuropsychiatric disorders at one month following TBI.

Variables			
Neuropsychiatric manifestations	Methods of Measurements	Operational definition	Level of measurements
Major Depressive Disorder	M.I.N.I and PHQ-9 questionnaire	Yes or No	Categorical
Generalized Anxiety Disorder	M.I.N.I and GAD-7 questionnaire	Yes or No	Categorical
PTSD	M.I.N.I and PCL-5	Yes or No	Categorical
Bipolar-Mania	MINI and Young Mania Rating Scale	Yes or No	Categorical
Psychosis	MINI and PANSS	Yes or No	Categorical
Apathy	Apathy Evaluation Scale(AES)	Yes or No	Categorical
Neurocognitive disorders	Montreal Cognitive Assessment (MoCA)	Yes or No	Categorical

*Aim 2 variables*. Address the factors associated with neuropsychiatric manifestation in TBI patients at one month, which include age, sex, occupation, education level, substance use, intoxication with alcohol during injury, severity of TBI, nature of injury, cause of trauma, area affected, type of lesion, duration of loss of LOC, duration of PTA, history of seizures (Table 2).

**Table 2:** Description of factors associated with neuropsychiatric manifestation at one-month post-TBI.

Variables	Methods of Measurement	Operational definition	Level of Measurements
Age	Patient’s file report	Age (years)	Continuous
Sex	Patient’s file report	Male or female	categorical
Occupation	Patient’s file report	Driver, footballer and others	categorical
History of substance use	Patient’s file report	Yes or no	categorical
History of being intoxicated with alcohol	Patient’s file report	Yes or no	categorical
**Clinical characteristics**			
Aphasia	Clinical assessment	Yes or no	categorical
Dysarthria	Clinical assessment	Yes or no	categorical
Confusion	Clinical assessment	Yes or no	categorical
Limb weakness/laterality	Clinical assessment	Yes or no	categorical
Seizures/ convulsions	Clinical assessment	Yes or no	categorical
**Severity of TBI**			
Glasgow coma scale	Patient’s file report	Moderate and severe	Categorical
Duration of LOC	Patient’s report	<30minutes, 30minutes to 24hrs >24hrs	categorical
Duration of PTA	Patient’s report	<24hrs 1day to 7days >7 days	categorical
**Injury characteristics**			
Nature of injury	Clinical assessment	Open, closed Sharp, blunt Penetrative, non-penetrative	categorical
Cause of trauma	Patient’s file report	RTA, falls, hit by object	categorical
Area affected	CT/MRI	Frontal, temporal, parietal, occipital	categorical
Type of lesion	CT/MRI	Hematoma, diffuse axonal injury, cerebral edema	categorical

*Aim 3 variables*. The variables addresses the cumulative incidence of neuropsychiatric disorders at three and six months following TBI, that is all neuropsychiatric manifestations identified between one and six months, including Depression, Generalized Anxiety Disorder, Bipolar, PTSD, Neurocognitive disorder, Psychosis and Apathy (Table 3).

**Table 3:** Description of cumulative incidence of NPS manifestations at one and six months post TBI.

Variables	Methods of measurement	Operational definition	Level of measurement
Any neuropsychiatric condition between one month and six months	MINI, PHQ-9, GAD-7, PCL5, MoCA, YMRS, AES, PANSS	Presence of any neuropsychiatric manifestation	Categorical

*Aim 4 variables*. The variables address predictors of any neuropsychiatric disorder at three and six months, which include age, sex, occupation, education level, substance use, intoxication with alcohol during injury, severity of TBI, nature of injury, cause of trauma, area affected, type of lesion, duration of loss of LOC, duration of (PTA), history of seizures, presence of any neuropsychiatric manifestation at one and three months, presence of seizures any time during follow up, medications given during follow up period (Table 4).

**Table 4:** Description of predictors of neuropsychiatric manifestations at three and six months post-TBI.

Variables	Methods of Measurement	Operational definition	Level of Measurements
Age	Patient’s file report	Age (years)	Continuous
Sex	Patient’s file report	Male or female	categorical
Occupation	Patient’s file report	Driver, footballer and others	categorical
History of substance use	Patient’s file report	Yes or no	categorical
History of being intoxicated with alcohol	Patient’s file report	Yes or no	categorical
**Clinical characteristics**			
Aphasia	Clinical assessment	Yes or no	categorical
Dysarthria	Clinical assessment	Yes or no	categorical
Confusion	Clinical assessment	Yes or no	categorical
Limb weakness/laterality	Clinical assessment	Yes or no	categorical
Seizures/ convulsions	Clinical assessment	Yes or no	categorical
**Severity of TBI**			
Glasgow coma scale	Patient’s file report	Moderate and severe	Categorical
Duration of LOC	Patient’s report	<30minutes, 30minutes to 24hrs >24hrs	categorical
Duration of PTA	Patient’s report	<24hrs 1day to 7days >7 days	categorical
**Injury characteristics**			
Nature of injury	Clinical assessment	Open, closed Sharp, blunt Penetrative, non-penetrative	categorical
Cause of trauma	Patient’s file report	RTA, falls, hit by object	categorical
Area affected	CT/MRI	Frontal, temporal, parietal, occipital	categorical
Type of lesion	CT/MRI	Hematoma, diffuse axonal injury, cerebral oedema	categorical
Neuropsychiatric manifestations at one and three months	MINI, PHQ-9, GAD-7, PCL5, MoCA, YMRS, AES, PANSS	Presence of any neuropsychiatric manifestation	Categorical
Presence of seizures during follow-up period	Patient’s report	Yes or no	categorical
History of using medications during follow-up	Patient’s report	Yes or no	categorical

### Data collection instruments

#### Mini International Neuropsychiatric Interview (MINI)

Will be used to diagnose psychiatric manifestations; it is a structured diagnostic interview using the DSM-5 and ICD-10 criteria [28]. We will administer module A(depressive disorder), module C (bipolar disorder), module H (PTSD), module N (Generalized anxiety disorder), and module K (psychosis). It is a useful tool for health workers in diagnosing psychiatric disorders and has an accuracy of 91.8% compared to ICD 10 [29]. MINI is also useful in identifying comorbidities, as shown in a study where a third of patients had ≥ 2 psychiatric diagnoses [30]. MINI, which has acceptably high validation and reliability scores and can also be administered in a much shorter time (mean 18.7 + 11.6 min, median 15 min), clinicians can use it after a brief training session, and lay interviewers require more extensive training [31].

#### Patient Health Questioner (PHQ-9)

Will be used to assess depressive symptoms with scores ranging from 0 to 27 since each of the nine items can be scored from 0 (not at all) to 3 (nearly every day) [32]. The items are; depressed mood, loss of interest in pleasurable activities, weight loss or weight gain, insomnia or hypersomnia, psychomotor agitation or retardation, fatigue or loss of energy, feeling of worthless or guilt, poor concentration and suicidal ideation, matching the Diagnostic and Statistical Manual of Mental Disorders, Fifth Edition (DSM-V) criteria of major depressive disorder [33]. The tool also measures depression severity with (5–9) categorized as mild, (10–14) as moderate, (15–19) moderately severe, (20–27) severe [34]. The likelihood of having a major depressive disorder is marked at PHQ score ≥ 15 [34] with sensitivity and specificity of 75% and 76%, respectively [35].

#### The Generalized Anxiety Disorder-7 (GAD-7)

A seven-item scale for generalized anxiety disorder, will be used to evaluate anxiety symptoms (feeling anxious, not being able to stop worrying, worrying too much about many things, trouble relaxing, being restless, being easily annoyed or irritable, feeling afraid as if something bad will happen) [36]. Response options are "not at all," "several days," "more than half the days," and "nearly every day," with scores of 0, 1, 2, and 3, respectively [36]. In clinical practice and research, GAD-7 is a reliable and accurate tool for screening GAD and measuring the severity of the disorder [36]. A cut-off point of 5 for mild, 10 for moderate and 15 for severe anxiety have demonstrated maximum sensitivity of 89% and specificity of 82% [36].

Will be used to assess manic symptoms (elevated mood, increased energy or motor activity, increased sexual interest, decreased need for sleep, being easily irritable over talkative, flight of ideas and grandiosity) [33]. It is an 11-item interviewer-rated scale [37]. The four items that are assessed on a 0–8 range are irritability, speech, thought content, and disruptive or violent conduct [37]. The remaining seven categories are graded on a 0–4 scale, with cut-off values of minimal (13), mild (20), moderate (26), and severe (38) manic symptoms [37]. It is a valuable instrument in screening patients with bipolar disorder in the manic phase with a cut-off point of 12.5, sensitivity of 93% and specificity of 96% [37].

#### Apathy Evaluation Scale (AES)

Will be used to assess apathy. It was created by Marin(1991) as a tool for assessing apathy brought on by pathologies of the brain [38]. AES is a reliable and accurate indicator of apathy following a TBI [39]. The scale has 18 items, each evaluated on a Likert scale with four possible responses, and it is measured on a scale from 18 to 72, with higher scores indicating greater apathy [40]. The optimal trade-off between sensitivity(83%) and specificity(67%) is provided by a score of 36 [41].

#### Positive and Negative Syndrome Scale (PANSS)

It is a scale used in medicine to rate the severity of symptoms in people with psychosis [42]. Seven Positive Scales, P(delusion, disorganized behaviour, hallucination, excitement, persecution/suspiciousness and hostility), seven Negative Scales, N(blunted affect, emotional withdrawal, poor rapport, passive, difficulty in abstract thinking, lack of spontaneity, and stereotyped thinking), the remaining sixteen are General Psychopathology Scales, G(somatic complain, anxiety, guilty feeling, tension, mannerism/ posturing, depression, motor retardation, uncooperativeness, unusual thought content, disorientation, poor attention, lack of judgment and insight, disturbance and volition, poor impulse control, preoccupation and active social avoidance) make up the remaining thirty items on the PANSS [43]. The optimum cut-off point of 38.5 has a sensitivity of 96% and specificity of 95.9% [43].

#### The PTSD checklist for DSM-5 (PLC-5)

Will assess PTSD symptoms. Similar to the PCL-C version [44], the PCL-5 item scores are added up to produce a continuous measure of the severity of PTSD symptoms using DSM-5 criteria for both individual symptom clusters and the entire condition [44]. Twenty items make up the PCL-5, each of which is scored on a five-point Likert-type scale, and the scores range from "Not at all" (zero) to "Extremely" (four), yielding a symptom severity score that ranges from 0 to 80 [45]. The cut-off score of 23 has achieved the optimal balance of sensitivity of 82% and specificity of 70% [46].

#### Montreal cognitive assessment

Will be used to evaluate cognitive functioning (MoCA). It is a rapid cognitive screening instrument with high sensitivity and specificity for identifying mild cognitive impairment [47]. MoCA assess visuospatial/executive function, which has 5 points, naming has 3 points, memory-5 points, attention-2 points, language-3 points, abstraction-2 points, and orientation 6 points [47]. Supported by a meta-analysis, a cut-off score of 23 will be used instead of the commonly used score of 26 [47]. Although the cut-off score of 23 decreases the sensitivity from 94% to 83%, specificity significantly improves from 66% to 88%, thus having an overall better diagnostic accuracy, even among the less educated population [48].

### Data analysis plan

All data will be analyzed using Statistical Analysis Software (SAS) 9.4. Descriptive statistics such as mean, median, frequency and standard deviation will describe the baseline characteristics of participants, and a logistic regression model will be run to determine the association between predictors and Neuropsychiatric outcomes at 1, 3 and 6 months. An Odd ratio > 1 will indicate a high likelihood that an independent variable is significantly associated with or without neuropsychiatric manifestations. Likewise, an odds ratio < 1 will indicate less likely that an independent variable is associated with NPS or no NPS manifestation.

Those with p-value less than 0.2 at univariate logistic regression analysis will be computed under multivariable analysis with a p-value set at <0.05 significance level.

### Ethical consideration

The ethical clearance has been secured from the Institutional Research Review Ethics Committee of the University of Dodoma with the reference number MA.84/261/12. The respective authorities provided permission to conduct the study within the premises of Benjamin Mkapa Hospital (AB.150/293/O1/360) and Dodoma Regional Referral Hospital (PB.22/1307/02/112).

The study participants or their relatives will be verbally informed of their participation in the study, and those with the capacity to provide informed consent for participation in the study will be recruited; otherwise, a close family member or custodian will give the consent on their behalf. The consent form will clearly state the benefit of participation in the study, including receiving medical and psychological treatments when needed. The informed consent document is translated into Swahili, the spoken language of most participants. The document will be read to those who cannot read it. All consenting subjects will sign the consent forms, and for those individuals who are unable to write, thumbprints will be used instead. The participants will be reassured that they are free to stop at any point during the study, and this will not affect their access to the services they routinely receive at these hospitals. Confidentiality will be assured and maintained. Patients diagnosed with any psychiatric illness will be referred for further assessment and treatment.

### Study timeline

This study will take 13 months, from October 2023 to November 2024, in which enrolment will be done for four to six months, and every patient will be followed for six months.

## Discussion

The longitudinal prospective nature of the study will offer a strong temporal association of the incidence, progression and associated factors of neuropsychiatric manifestations post-TBI. The study excluded patients with a known history of psychiatric diagnosis that could influence the recurrence relapse of the present illness and the existence of another new psychiatric diagnosis after TBI.

The diagnostic tool "International Neuropsychiatric Interview (MINI) " is highly reliable and sensitive as it utilizes both DSM and ICD criteria [28] and screening tools which will be used have good validity and reliability in assessing the severity progression of neuropsychiatric symptoms.

Since there is minimal information related to the subject area, only two studies have been published in sub-Saharan African settings [23, 24]; this study will offer needed data regarding the incidence and predictors of post-TBI neuropsychiatric manifestations in our settings.

The final results will be submitted to the University of Dodoma library, the study areas (Dodoma Regional Referral Hospital and the Benjamin Mkapa Hospital), and the manuscripts will be ready for submission to different peer-reviewed journals before publication.
